# Influences of human thorax variability on population rib fracture risk prediction using human body models

**DOI:** 10.3389/fbioe.2023.1154272

**Published:** 2023-03-23

**Authors:** Karl-Johan Larsson, Johan Iraeus, Sven Holcombe, Bengt Pipkorn

**Affiliations:** ^1^ Autoliv Research, Vårgårda, Sweden; ^2^ Division of Vehicle Safety, Department of Mechanics and Maritime Sciences, Chalmers University of Technology, Gothenburg, Sweden; ^3^ International Center for Automotive Medicine, University of Michigan, Ann Arbor, MI, United States

**Keywords:** human body model (HBM), rib fracture, sensitivity analysis, cortical bone, rib material

## Abstract

Rib fractures remain a common injury for vehicle occupants in crashes. The risk of a human sustaining rib fractures from thorax loading is highly variable, potentially due to a variability in individual factors such as material properties and geometry of the ribs and ribcage. Human body models (HBMs) with a detailed ribcage can be used as occupant substitutes to aid in the prediction of rib injury risk at the tissue level in crash analysis. To improve this capability, model parametrization can be used to represent human variability in simulation studies. The aim of this study was to identify the variations in the physical properties of the human thorax that have the most influence on rib fracture risk for the population of vehicle occupants. A total of 15 different geometrical and material factors, sourced from published literature, were varied in a parametrized SAFER HBM. Parametric sensitivity analyses were conducted for two crash configurations, frontal and near-side impacts. The results show that variability in rib cortical bone thickness, rib cortical bone material properties, and rib cross-sectional width had the greatest influence on the risk for an occupant to sustain two or more fractured ribs in both impacts. Therefore, it is recommended that these three parameters be included in rib fracture risk analysis with HBMs for the population of vehicle occupants.

## 1 Introduction

While the general injury risk for vehicle occupants in crashes has been reduced over time, the risk for sustaining rib fractures remains high (Forman et al., 2019; Kullgren et al., 2020; Pipkorn et al., 2020). Generally, crash injury statistics indicate that the risk for thoracic injuries and rib fractures increases with advancing age and increasing body mass index (BMI), and is greater for females than for males (Bose et al., 2011; Carter et al., 2014; Forman et al., 2019). These differences indicate that further consideration of occupant variability in the design and evaluation of vehicle safety is important for reducing rib fracture risk in crashes.

Improved occupant protection has been achieved through vehicle safety development using anthropomorphic test devices (crash test dummies) as occupant substitutes in both physical and computer modeled crash tests. These dummies have simplistic representations of the human anatomy and are used to estimate occupant injury risk through body region assessments in crash tests; for example, chest injury risk can be estimated based on chest compression. There are, however, indications that these estimations have only a limited capability to predict vehicle occupant rib fractures in real-world crashes (Kent et al., 2003; Brumbelow, 2020; Brumbelow et al., 2022).

As a complement to crash test dummies, finite element human body models (HBMs), such as THUMS (Shigeta et al., 2009), GHBMC (Gayzik et al., 2012), SAFER HBM (Pipkorn et al., 2021), and VIVA+ (John et al., 2022) are also used in vehicle safety research and development. These computational models of human occupants have detailed ribcage modeling, including representations of the individual ribs. Rib fracture injury can be evaluated at the tissue level using measurements physically related to fracture, such as strain in the rib cortical bone (Trosseille et al., 2008). In the THUMS and GHBMC HBMs, rib elements that have reached a pre-defined strain threshold are deleted, and deleted elements are considered to represent fractures in the rib. Alternatively, a probabilistic framework proposed by Forman et al. (2012) utilizing an age-adjusted strain-based fracture risk function, has been used for rib fracture risk predictions with the SAFER HBM (Pipkorn et al., 2019). Using this method, age-adjusted risks of rib fractures are calculated using the maximum strain from each rib. Previous validation of HBM rib fracture prediction has shown that the SAFER HBM (v.9) predicted the rib fracture outcomes from sled tests with *post mortem* human subjects (PMHS) and from accident reconstructions with good accuracy. However, in a stochastic study, the predicted rib fracture risk for 30-year-old occupants was too high (Pipkorn et al., 2019; Larsson et al., 2021). On the other hand, studies with THUMS (AM 50 v.4) and GHBMC (M50 v.4.2) have predicted fewer rib fractures than those sustained by PHMS' in corresponding impact conditions (Shigeta et al., 2009; Schoell et al., 2015).

An inherent difficulty with predicting the rib fracture outcomes in impact experiments or real-word crashes is that the outcome depends on individual factors that are difficult to control for. While age, sex, height, and weight are commonly controlled for, material mechanical properties and local geometry are not, although they potentially contribute to the occurrence of rib fractures as well. Studies of these factors have reported large individual differences. Single rib and overall ribcage geometry, rib cross-sectional dimensions, and rib cortical bone thickness vary between individuals (Holcombe et al., 2017a; Holcombe et al., 2019). In impact experiments with single ribs, it was found that cross-sectional geometrical measures taken adjacent to the fracture location (such as total and cortical bone area and cortical bone thickness) and area moment of inertia explain some of the variability in rib stiffness and fracture force (Murach et al., 2017; Agnew et al., 2018; Liebsch et al., 2021). Rib cortical bone material parameters, such as yield stress and failure strain, trend toward declining values with increasing age, but nevertheless show a substantial variability between individuals of similar age (Katzenberger et al., 2020; Velázquez-Ameijide et al., 2021). Individual variability exists in the rib trabecular bone (Kemper et al., 2020) and in the costal cartilage connecting the ribs to the sternum (Forman et al., 2010) as well. Experiments with human and animal samples also demonstrate variability in soft tissue mechanical characteristics, for both skeletal muscle and adipose tissue (Van Sligtenhorst et al., 2006; Gefen and Haberman, 2007; Böl et al., 2012; Sommer et al., 2013; Sun et al., 2021).

Aggregated, these results suggest that individual variability produces variable rib fracture outcomes—even in controlled PMHS experiments. The following examples lend further support to this finding. First, in a group of eight PMHS’ that all had a peak chest compression of 28% ± 1% in frontal chest impact experiments, between zero (two subjects) and 17 (one subject) rib fractures were sustained (Kent and Patrie, 2005). Second, in frontal sled tests with five reclined and belted PMHS’, between zero and 22 rib fractures were sustained by the test subjects (Richardson et al., 2020). Third, in 3 m/s side impact sled tests, one out of seven subjects sustained six rib fractures, while three sustained zero (Miller et al., 2013). Thus, due to the inherent variability, it is reasonable to expect a distribution of rib fracture outcomes when different individuals are subjected to the same impact scenario.

In order to design vehicles and safety systems with reduced or mitigated rib fracture risk for vehicle occupants, knowledge of the injury distribution from HBM simulations, and how it is affected by design alterations, is valuable. Traditionally, an HBM is a fixed representation of a single individual, often with material and geometrical properties representing an average person from a particular subpopulation, such as a 50th percentile male or a fifth percentile female (in height and weight). Therefore, the HBM will predict a single fixed rib fracture outcome in an impact simulation. An exception is probabilistic rib fracture risk prediction, which can produce multiple age-adjusted risk predictions from the same, fixed, HBM rib strain predictions. In recent years, morphing (re-shaping) the geometry of HBMs based on statistical human shape models has been used to create several HBMs that geometrically represent male and female occupants of varying age, height, and weight (Hu et al., 2019; von Kleeck et al., 2022; Larsson et al., 2022b). However, these HBMs still represent geometrically average individuals, with an average ribcage shape, for the subpopulation described by each choice of sex, age, height, and weight. However, ribcage shape statistical models based on these parameters can only explain approximately 50% of human ribcage shape variability (Wang et al., 2016; Holcombe et al., 2017a). The residual variability in ribcage shape is potentially important for assessing rib fracture risk.

A general limitation with models created with average inputs is that they do not necessarily predict the average outcome if non-linearities are present (Cook and Robertson, 2016). As rib fracture risk is non-linear in terms of strain (Larsson et al., 2021) any effects on strain in the ribs from geometrical and material variations have non-linear effects on rib fracture risk. Therefore, it cannot be known if the rib fracture prediction obtained from a single HBM representing some subpopulation average is an over- or underestimation; there is a need for more knowledge about the injury distribution in order to make informed design choices. A recent study presented a methodology to compute distributions of probabilistic rib fracture risks through HBM simulations in a far-side crash scenario (Perez-Rapela et al., 2021). In that study, a response surface (i.e., a meta-model) was created to predict HBM rib fracture risk based on six parameters describing human, crash, and safety system variability. The final response surface, created from 405 input-output examples, was then used in Monte Carlo simulations to compute the distributions of rib fracture risk as safety system parameters were altered. The human variability parameters (used to morph the HBM for every simulation) were height, weight, and waist circumference, but no parameters representing human variability in material properties or rib and ribcage geometry were included. Therefore, the effect of these parameters on rib fracture risk is not known.

A major hurdle for including these aspects of human variability in vehicle safety evaluations with HBMs is the exponential growth in the number of possible parameter combinations as the number of parameters increases. As vehicle crash simulations with HBMs are computationally expensive (hours to days per simulation, depending on the specific load case and computing resources), it is important to minimize the number of model parameters. To facilitate an informed tradeoff between computational cost and the information gained about the potential injury outcomes, it is necessary to know which human variability factors to prioritize for inclusion. Further, detailed anatomical and biomechanical reference data needed for HBM building are limited. Knowledge about which variability inputs are most important can provide guidance for future anatomical and biomechanical characterization studies. Therefore, the aim of this study was to identify the human thorax property variations that influence rib fracture risk for the population of vehicle occupants in two crash scenarios.

## 2 Materials and methods

The study was carried out in two steps. The first step was to represent individual variability by parametrizing existing geometry and material models of the SAFER HBM v10 (SHBM) (Pipkorn et al., 2021). The SHBM was chosen as the baseline HBM for the study because it has a ribcage model validated for rib cortical bone strain and strain-based probabilistic rib fracture risk predictions in various impact configurations (Iraeus and Pipkorn, 2019; Pipkorn et al., 2019).

In the second step, the parametrized SHBM was subjected to frontal and near-side impact scenarios in generic vehicle interior sled models (Iraeus and Lindquist, 2016; Pipkorn et al., 2019). A parametric sensitivity analysis was performed to quantify how the variability of certain geometrical and material parameters contributed to rib fracture risk predictions. Rib fracture risk, the risk that an occupant sustained two or more fractured ribs (NFR2+), was calculated using the age- and strain-based probabilistic method (Forman et al., 2012; Larsson et al., 2021). The maximum of first principal strain in each rib cortical bone, calculated in the middle element layer, was used for the risk calculation. The occupant age was fixed at 45 years in the NFR2+ calculations, as this corresponds to a rib fracture risk function of roughly average strain sensitivity across the age span of bone samples it was constructed from (Larsson et al., 2021).

All simulations were performed using LS-Dyna (16 cores, R9.3.1 MPP, Livermore Software Technology, Livermore California, United States.)

### 2.1 Representing human variability through HBM parametrization

The geometric features of the ribs and ribcage and material models of the SHBM, detailed in the following sections, were parametrized to represent the population variability (sourced from published studies). As the SHBM represents an average male occupant, male data were used where applicable.

Each parametric variation was driven by a scaling coordinate, s, which either corresponds to the number of standard deviations (SDs) or was interpolated in a range defined by upper and lower bounds (depending on available data for the parameter). The targeted range of variability for each parameter was ±2 SDs, or 95% of the range of available data. The exceptions were costal cartilage modulus and material properties for muscle and adipose tissue: the costal cartilage modulus was varied within a range corresponding to 90% of the estimated distribution due to instabilities in the costal cartilage elements for low modulus values; for muscle and adipose tissue, the upper and lower bounds of material parameters were based on tissue behavior in different test setups.

#### 2.1.1 Rib and ribcage geometry

Ribcage shape, rib cross-sectional dimensions, and rib cortical bone thickness were parametrized according to the expressions summarized in Table 1. The residual variability in ribcage shape, i.e., the variability not explained by sex, age, height, and weight trends in statistical ribcage shape models, was modelled through representing the ribcage shape variability among a sample of average males. Parametrization of ribcage shape, previously presented in Larsson et al. (2022a), was based on principal component analysis (PCA) of ribcage geometric data from average height and weight males. The process is briefly explained below.

**TABLE 1:** Parameters modifying rib and ribcage geometrical features and the parametric expressions used.

Parameter name	Parametric expression
Ribcage shape PC’s, i=1,…,6	$\begin{array}{c} C_{i}\left(s\right)=\mu +P_{i}^{T}{*\sigma }_{i}*s \end{array}$ (*Eq*.1)
Rib cortical bone thickness	$\begin{array}{c} T\left(s\right)=\exp\left(\log\left(T_{nom}\right)+\sigma_{\mu }*s\right)\;\left[mm\right] \end{array}$ (*Eq*.2)
Rib cross-sectional width	$\begin{array}{c} W\left(s\right)=W_{nom}+\frac{2.7}{2}*s\;\left[mm\right] \end{array}$ (*Eq*.3)
Rib cross-sectional height	$\begin{array}{c} H\left(s\right)=H_{nom}+\frac{3.9}{2}*s\;\left[mm\right] \end{array}$ (*Eq*.4)

Parametric curves describing rib centroidal path geometry (curves passing through the centroid of consecutive rib cross-sections), previously fitted to CT-scan data of over 1,000 individuals (Holcombe et al., 2016; Holcombe et al., 2017a; Holcombe et al., 2017b), were used to geometrically describe ribcage shape. From these individuals, n = 89 males were chosen based on the inclusion criteria: age >18 years, height 1.72–1.82 m, and weight 72–82 kg. Next, a PCA was performed using points generated along the n = 89 sets of rib curves. The first six of the resulting principal components (PCs) together described more than 90% of the variance in ribcage shape, so they were used to morph the SHBM ribcage and surrounding parts to represent variability in ribcage shape.

**FIGURE 1:**
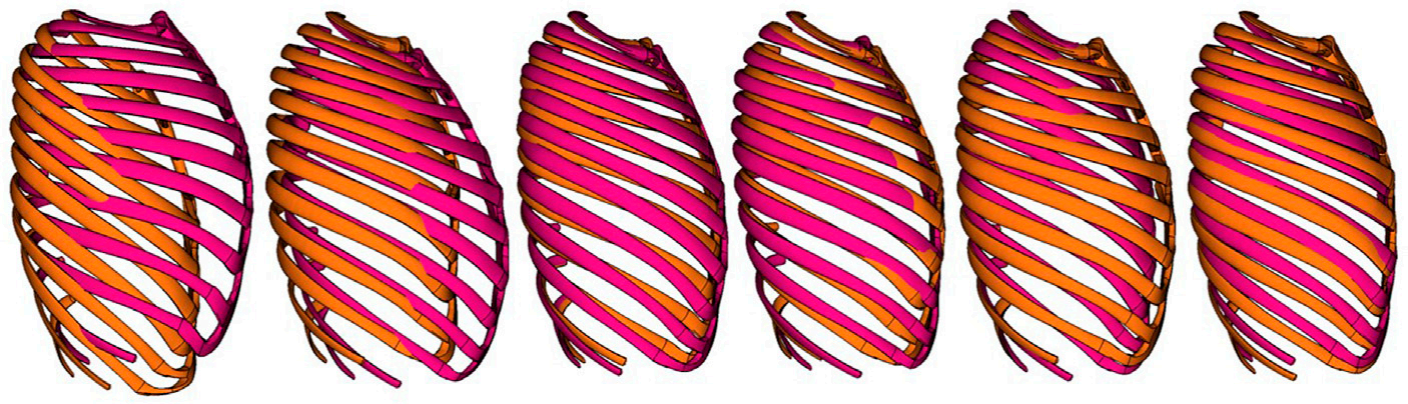
Lateral view of ribcage of SHBM as morphed to ±2 SDs of corresponding scores for PC 1–6 (left to right).

**FIGURE 2:**
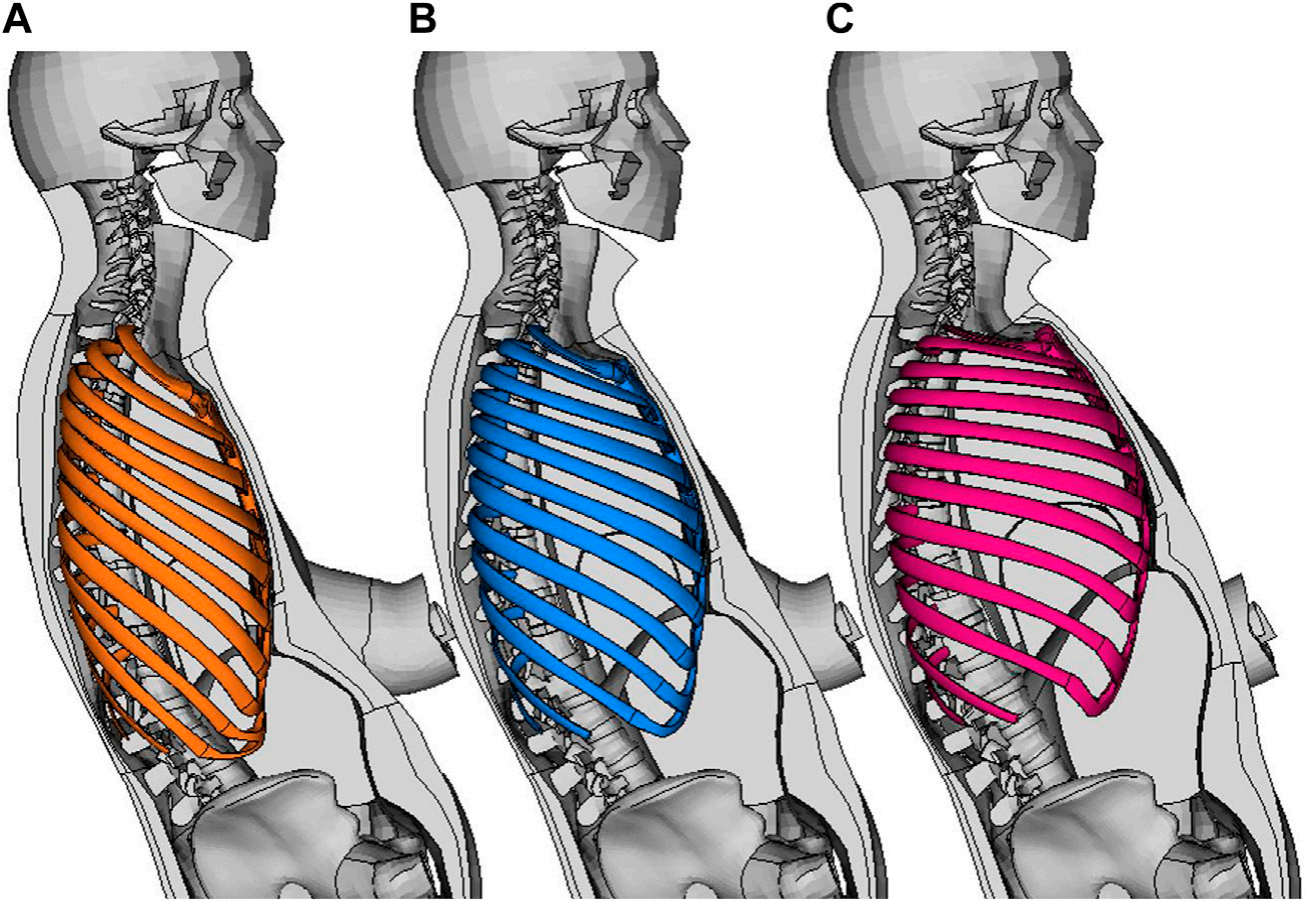
SHBM torso with parts removed to show ribcage and internal parts. **(A)**: Ribcage morphed to −2 SDs of PC 1 score, **(B)**: 0 (average ribcage shape), **(C)**: +2 SDs for PC 1 (left to right).

For each PC, morphing targets were generated by the parametric expression in Eq. 1, where $C_{i}$ is a vector of rib curve point coordinates, μ is the average point coordinate from PCA, $P_{i}$ is the i th principal component, $\sigma_{i}$ is the sample SD of PC scores for the i th principal component, and s is a scaling coordinate. Thus, for i=2 and s=1.5 , $C_{i}\left(s=1.5\right)$ represents the discretized rib curves of a ribcage with 1.5 SD of the score for PC 2. First, the morphing aligned the centroidal paths of the SHBM ribs to the corresponding centroidal paths generated by Eq. 1, and second, it adapted surrounding tissues to the changes in ribcage shape. Ribcages morphed to ±2 SDs of the sample scores for each PC are shown in Figure 1. The SHBM torso, as morphed to adapt to the ribcage shape changes from PC 1, is shown in Figure 2. Further details of PCA and SHBM morphing are provided in Larsson et al. (2022a).

**FIGURE 3:**
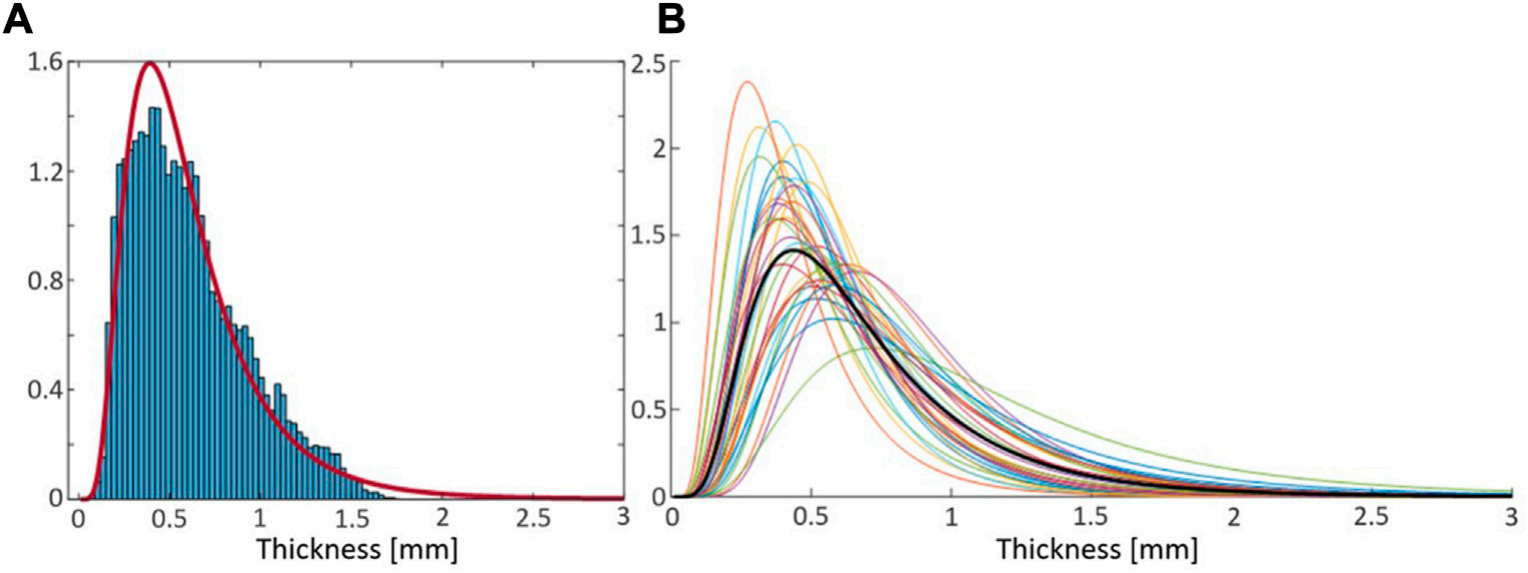
**(A)**: Density histogram of all rib cortical bone thickness measurements from a representative individual (blue bars) and probability density function of the corresponding fitted log-normal distribution (red curve). **(B)**: Probability density functions of fitted log-normal distributions from all 33 individuals in Holcombe et al. (2019), and the distribution from mean of fitted parameters (thick black curve).

The SHBM has varying rib cortical bone thickness, along and around all ribs, assigned at each node in the rib element meshes, based on averaged male measurements (Choi and Kwak, 2011). To determine scaling ranges for the thickness, individual maps of rib cortical bone thickness from Holcombe et al. (2019) were re-analyzed. Within an individual rib, the thickness measurements were approximately log-normally distributed (Figure 3). Thus, parametric log-normal distributions with parameters μ and σ were fitted to all 33 individual rib measurements (Figure 3). In log-space, the fitted μ parameters were approximately normally distributed, with an SD of $\sigma_{\mu }=0.22$ ; this value was used to scale each nodal thickness value of all the SHBM ribs according to Eq. 2, where $T\left(s\right)$ is the new nodal thickness value, $\exp\left(\right)$ and $\log\left(\right)$ are the natural exponential and logarithm functions, and $T_{nom}$ is the original nodal thickness value. The same scaling factor was used to scale the thickness all 24 ribs simultaneously.

The SHBM ribs have elliptical cross-sections, with dimensions as well as cross-sectional orientation varying along the ribs and between rib levels, based on average male measurements (Choi and Kwak, 2011; Iraeus et al., 2020). Variability in rib cross-sectional height and width was achieved by morphing SHBM ribs along the major (rib height) and minor (rib width) axes of the elliptical rib cross-sections. To determine scaling ranges, SDs of maximum and minimum area moments of inertia ( $I_{\max}$ and $I_{\min}$ ) of male sixth-level ribs were used (Holcombe et al., 2019). As the SHBM has simplified representations of rib sternal and vertebral ends, the average SD values for only 20%–80% of the rib span length were used. The cross-sections of all ribs were scaled simultaneously along the entire length of the ribs, using the same scaling ranges around their respective nominal width, $W_{nom}$ (Eq. 3; Table 1), or height, $H_{nom}$ (Eq. 4; Table 1), using the Ansa pre-processor (v19.1, Beta CAE Systems, Thessaloniki, Greece). In Table 1, Eqs 3, 4, a cross-sectional height scaling of ±3.9 mm corresponds to an average rib area moment-of-inertia change of ±2*SDs of $I_{\max}$ , and a width scaling of ±2.7 mm corresponds to ±2*SD of $I_{\min}$ . In a subsequent step, the surrounding soft tissues were morphed, to ensure smooth transitions to the intercostal muscle mesh connected to the ribs and to avoid contact surface intersections as rib dimensions were altered.

To avoid the influence of torso mass variability on the rib fracture risk predictions, the density of soft tissue materials in the HBM were uniformly scaled to retain the original HBM mass for all parametric changes that modified the torso geometry.

#### 2.1.2 Variability in material mechanical parameters

**FIGURE 4:**
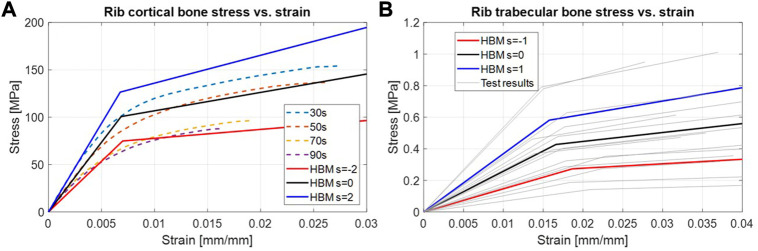
HBM lines represent the different values of the scaling coordinate used in the bi-linear material model. **(A)**: Rib cortical bone stress vs. strain. The range of 30–90s (dashed) is average responses for corresponding ages (Katzenberger et al., 2020). **(B)**: Rib trabecular bone stress vs. strain. Test results (gray lines) correspond to the material parameters from individual test results.

Table 2 summarizes the parametric expressions used to scale the material parameters of rib cortical bone, rib trabecular bone, and costal cartilage. For rib cortical bone, an isotropic bi-linear material model (LS-Dyna *MAT_24) was used (Iraeus et al., 2020). The material parameters Young’s modulus, E , yield stress, $\sigma_{Y}$ , and plastic modulus, P (Eq. 5) were co-varied to represent a “stiffer” or “softer” material response (by increasing or decreasing s , respectively). Figure 4 shows the bi-linear stress-strain curves for different levels of the scaling coordinate and compares them to a decade of average age curves from tensile testing at 0.5 strain/s (Katzenberger et al., 2020).

**TABLE 2:** Parametric expressions for scaling material model parameters of ribs and costal cartilage.

Parameter name	Parametric expression
Rib cortical bone	$\begin{array}{c} Cort\left(s\right)=\left\{\begin{array}{c} E=14.7+2.0*s\;\left[GPa\right]\\ \sigma_{Y}=100.7+12.9*s\;\left[MPa\right]\\ P=1.94+0.5*s\;\left[GPa\right] \end{array}\right. \end{array}$ (*Eq*.5)
Rib trabecular bone	$\begin{array}{c} Trab\left(s\right)=\left\{\begin{array}{c} E=25.7+46.7*\frac{0.95*s}{4}\;\left[MPa\right]\\ \sigma_{Y}=0.42+0.65*\frac{0.95*s}{4}\;\left[MPa\right]\\ P=5.66+11.9*\frac{0.95*s}{4}\;\left[MPa\right] \end{array}\right. \end{array}$ (*Eq*.6)
Costal Cartilage	$\begin{array}{c} Cart_{-}E\left(s\right)=21.4+1.15{*s}^{2}+9.05*s\;\left[MPa\right] \end{array}$ (*Eq*.7)

The SHBM rib trabecular bone was also modeled as a bi-linear isotropic material (LS-Dyna *MAT_24). The bi-linearity was based on material properties reported from compressive testing of human rib trabecular bone from 15 individuals (Kemper et al., 2020). The expression (Eq. 6) scaling the trabecular bone material parameters was configured to cover 95% of the range of material parameters for the scaling coordinate $s\in \left[-2,2\right]$ .

The costal cartilage was updated to use the effective material modulus identified from 28 individuals (Forman et al., 2010). For $s\in \left[-2,2\right]$ , the expression in Eq. 7 interpolates the 5th to 95th percentiles of a log-normal distribution with the sample mean and SD of 21.4 ± 12.0 MPa from Forman et al. (2010).

The SHBM subcutaneous adipose tissue and skeletal muscle were modeled as visco-hyperelastic materials (LS-Dyna *MAT_077_O with Prony series). For adipose tissue, material parameters were varied together to represent “softer” and “stiffer” material representations; see Table 3. Parameter ranges were obtained through parameter identification (Naseri, 2021) for tissue samples in different test setups (Gefen and Haberman, 2007; Geerligs et al., 2008; Comley and Fleck, 2012). The muscle tissue material model was used for both the thoracic skeletal muscles and the intercostal muscles. The nominal material was presented by Lanzl et al. (2021). The bulk modulus was varied for the muscle material across a range based on passive muscle cross-fiber compressive test results (Van Sligtenhorst et al., 2006; Böl et al., 2012; Mohammadkhah et al., 2016); see Table 3. As the parameter ranges for adipose and skeletal muscle tissue material models represent variability in tissue response across different experiments, rather than between individuals in a single experiment, the scaling parameters for these tissues were considered uniformly distributed in the following sensitivity analysis. This corresponds to an equal weighting of soft tissue material parameters, regardless of scaling coordinate value.

**TABLE 3:** LS-dyna *MAT_077_O material parameters for adipose and muscle tissues. Nominal, maximum, and minimum values and the parametric expressions used to vary the material parameters.

Material parameter	Nominal	Max	Min	Parametric expression for $s\in \left[-2,2\right]$
Adipose tissue				
Poisson’s ratio, ν [-]	0.49998	0.499995	0.49978	$\nu =0.499996-1.6216\exp\left(-1.2951s\right){*10}^{-5}$
µ [Pa]	35	41	29	µ=35+3*s
α [-]	20	20	20	
Viscoelastic Prony series				
$\beta_{i}$ [1/ms]	$G_{i}$ [kPa]			
0.006	0.80	1.04	0.56	$G_{1}=0.80+0.12*s$
0.05	1.80	2.34	1.26	$G_{2}=1.80+0.27*s$
0.6	2.22	2.90	1.54	$G_{3}=2.22+0.34*s$
Muscle tissue				
Poisson’s ratio, ν [-]	0.495	0.495	0.495	
µ [Pa]	108	63	153	µ=108+22.5*s
α [-]	13.2	13.2	13.2	

### 2.2 Parametric sensitivity analysis

Parameter influence on NFR2+ risk was quantified through a variance-based sensitivity analysis method (Zhang and Pandey, 2014). Using this method, for each parameter, sensitivity indices which quantified the contribution of input parameter variability to the total variance of the NFR2+ output were calculated. The method and variance-based sensitivity analysis is briefly described below.

The output of a model, Y , depends on its input parameters, $X=\left[x_{1},x_{2},...,x_{n}\right]$ , through some function, $Y=h\left(X\right)$ . For the current application, the model output was the NFR2+ rib fracture risk and the input parameters were those presented in the previous sections. The function was the occupant crash simulation and the NFR2+ risk calculation that resulted in an NFR2+ risk prediction for every configuration of the input parameters. All parameter values were considered normally distributed, except costal cartilage modulus, which was log-normally distributed, and soft tissues (adipose and skeletal and intercostal muscle material), which were considered uniformly distributed.

Variance-based sensitivity analysis utilizes the variance decomposition of the output (Sobol, 1990; Saltelli et al., 2008) (Eq. 8):

$$\begin{array}{c} V_{Y}=\sum_{i}^{n}V_{i}+\sum_{i}^{n}\sum_{j>i}^{n}V_{ij}+...+V_{ij...n} \end{array}$$ (8)

Where $V_{i}$ is the partial variance of Y due to varying parameter $x_{i}$ , $V_{ij}$ is due to the interaction of $x_{i}$ and $x_{j}$ , *etc.* The primary, or first-order, sensitivity index, defined as $S_{i}=\frac{V_{i}}{V_{Y}}$ , represents the main average effect contribution (disregarding interactions) of varying $x_{i}$ , for all possible combinations of the remaining input parameters. The second-order index is defined as $S_{ij}=\frac{V_{ij}}{V_{Y}}$ , and higher order sensitivity indices are defined analogously. The total sensitivity index; $S_{Ti}$ , accounts for the total contribution to $V_{Y}$ due to $x_{i}$ , including all higher-order interactions (Homma and Saltelli, 1996; Sobol, 2001).

The sensitivity indices can be calculated analytically for simple functions or be computed through Monte Carlo methods, sampling a large number of points X, for general functions. For the current study, sensitivity indices were calculated by an approximative method based on a multiplicative dimensional reduction method (M-DRM) (Zhang and Pandey, 2014). It is assumed that the model output around a chosen point in the input space, the cut-point: $X=C=\left[c_{1},c_{2},...,c_{n}\right],h_{0}=h\left(C\right)$ , can be decomposed into a set of one-dimensional functions, through M-DRM (Eq. 9):

$$\begin{array}{c} h\left(X\right)\approx h_{0}^{1-n}*\prod_{i=1}^{n}h_{i}\left(x_{i},C_{-i}\right) \end{array}$$ (9)

Where $h_{i}\left(x_{i},C_{-i}\right)$ is a function of $x_{i}$ and $C_{-i}$ is C without $c_{i}$ . From this assumption, it follows that computing one-dimensional integrals (through, e.g., Gaussian quadrature) provides sufficient information to calculate the sensitivity indices. For a function of n parameters and a quadrature rule of $N_{GP}$ Gauss points, at most $n*\;N_{GP}$ function evaluations are needed, see Zhang and Pandey (2014) for details.

Here, the cut-point, or the baseline case about which all parameters were varied, was selected with their average values (i.e., s=0 for all parameters), and a five-point Gauss-Legendre quadrature was used. The range of parameter variation considered was $s\in \left[-2,2\right]$ for all parameters; their values were modified according to the expressions presented in the previous sections. The parametric sensitivity analysis was performed twice for two different crash scenarios, a frontal impact and a near-side impact.

### 2.3 Occupant crash simulations

The SHBM was positioned as a driver in generic driver-side vehicle interior models equipped with generic representations of safety systems and the capability to model intrusions into the occupant compartment (Iraeus and Lindquist, 2016; Pipkorn et al., 2019). The vehicle models were subjected to accelerations corresponding to either a frontal impact or a near-side impact scenario. In the frontal impact scenario, the delta velocity was 45 km/h. The steering wheel airbag had a peak pressure of 25 kPa, and the pre-tensioned seatbelt was load-limited to 3.5 kN (as measured in the webbing above the HBM shoulder). The seatbelt was routed over the SHBM torso and lap using the closest path method in Primer pre-processor (v17.0 Oasys Ltd., Solihull, United Kingdom). No intrusion into the occupant compartment was modeled in the frontal impact case. The near-side impact had a peak door intrusion of 88 mm (measured at the armrest of the door panel interior), and a lateral delta velocity of 24 km/h. The seat-mounted side airbag had a peak pressure of 55 kPa, and the inflatable curtain had a peak pressure of 60 kPa. In both impacts, the delta velocity was chosen such that the cut-point version of the HBM predicted close to 50% risk of NFR2+. The vehicle and impact parameters were held constant in all subsequent simulations while the parameters representing human variability were varied in the HBM.

## 3 Results

**FIGURE 5:**
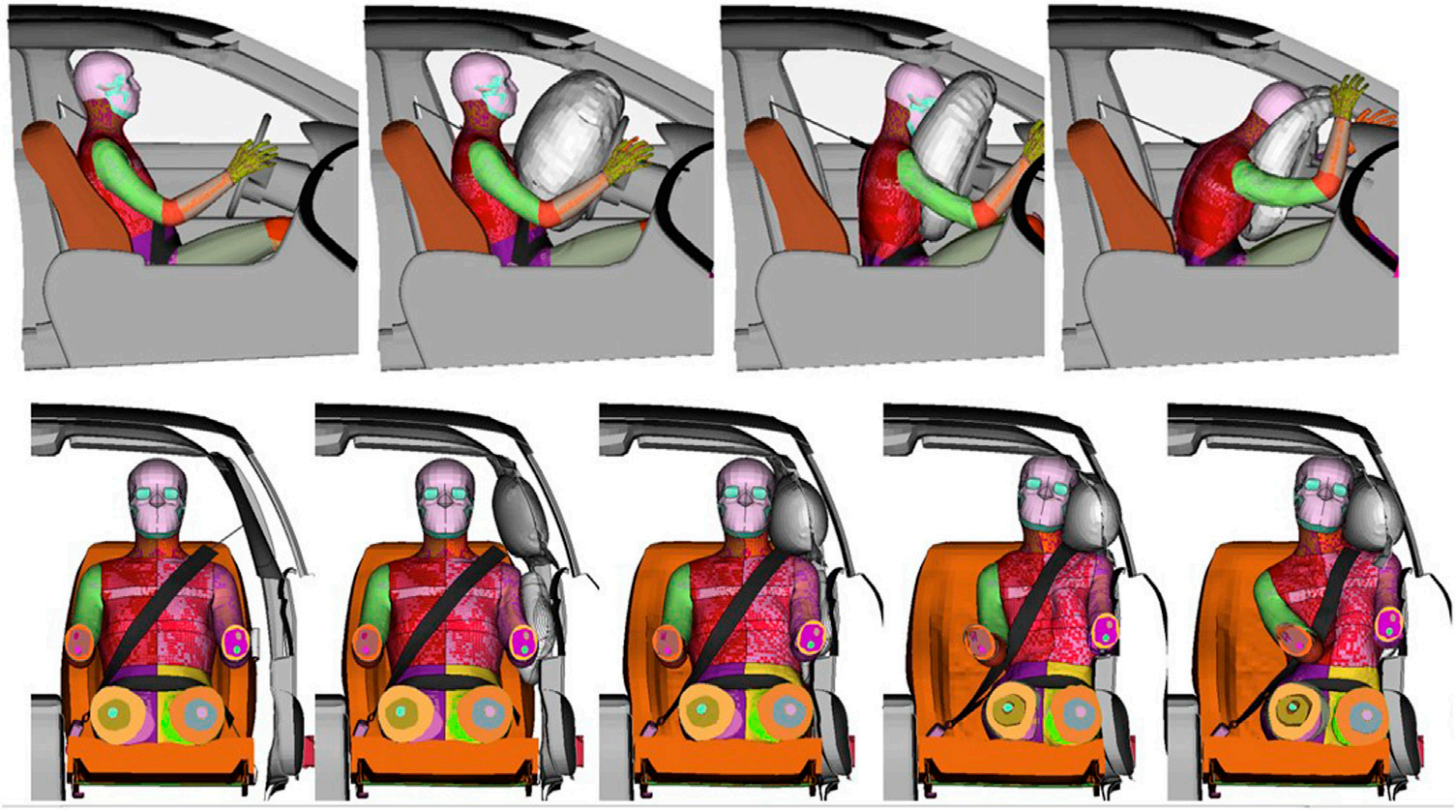
Kinematics of the SHBM in frontal and near-side lateral cut-point evaluations; s = 0 for all parameters. Top: Frontal impact at (left to right) 0, 40, 80, and 120 ms post-impact. Bottom: near-side impact at times 0, 20, 40, 60, and 80 ms post-impact. Arms and legs removed for improved visibility.

All simulations completed successfully (no error terminations). The resulting kinematics of the baseline SHBM with all parameters at their nominal values ( s=0 for all parameters) in the frontal and the near-side crash scenarios are shown in Figure 5. In both impact cases with the baseline HBM, the predicted NFR2+ risk was 51% (Supplementary Material; Supplementary Table SA1).

**FIGURE 6:**
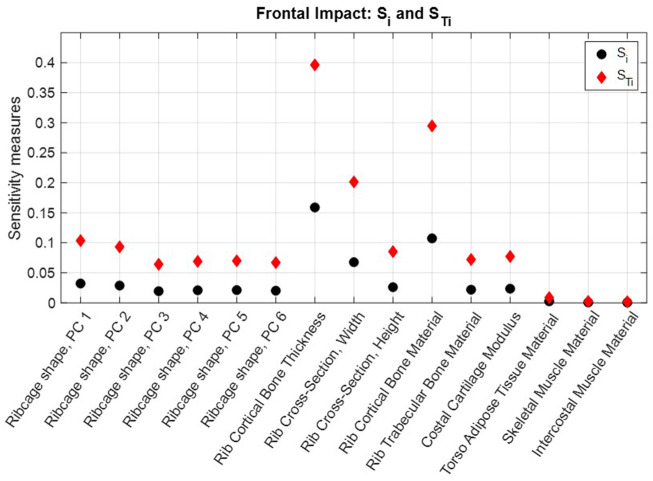
Frontal impact NFR2+ first-order sensitivity indices $S_{i}$ (black dots) and total sensitivity indices, $S_{Ti}$ (red diamonds) calculated for each parameter.

**FIGURE 7:**
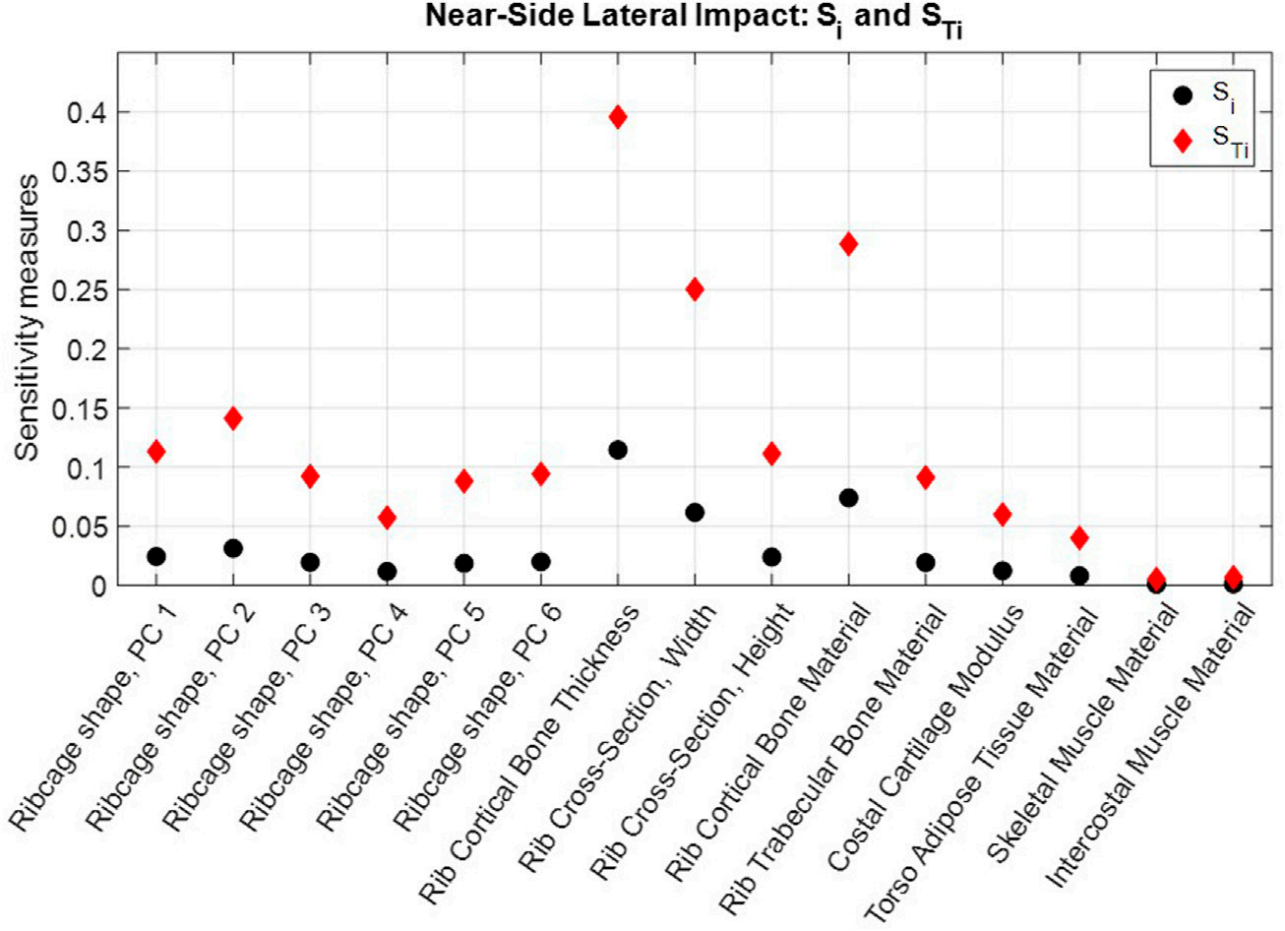
Near-side impact NFR2+ first order sensitivity indices, $S_{i}$ (black dots), and total sensitivity indices, $S_{Ti}$ (red diamonds), calculated for each parameter.

The NFR2+ risk predictions obtained in each evaluated scaling coordinate for the frontal impact and the near-side impact are shown in Supplementary Table SA1. First-order and total sensitivity indices are shown in Figure 6 for the frontal impact and Figure 7 for the near-side impact. In both impacts, rib cortical bone thickness, rib cross-sectional width, and rib cortical bone material properties were identified as the most influential for NFR2+ risk. Parameters representing soft tissue materials, i.e., torso adipose tissue, skeletal muscle tissue, and intercostal muscle tissue, had only a small influence on the NFR2+ risk in both impacts.

According to the total sensitivity indices, $S_{Ti}$ , approximately 40% of the variance in NFR2+ risk in both impact scenarios can be attributed to the population variability in rib cortical bone thickness alone. That is, for a fixed cortical bone thickness, the total variance of NFR2+ is reduced by 40%. The corresponding variance reductions for fixed rib cortical bone material parameters and fixed rib cross-sectional widths are 30% and 20%–25% (depending on impact), respectively (Figures 6, 7).

The differences between the first-order effects, $S_{i}$ , and the total effects, $S_{Ti}$ , for rib cortical bone thickness, rib cross-sectional width, and rib cortical bone material (Figures 6, 7) indicates the presence of substantial interaction effects for NFR2+. Second-order indices, $S_{ij}$ , for frontal impact are shown in Supplementary Figure SA1. Similar interaction effect magnitudes were obtained in both impacts. The three most influential interactions were between cortical bone thickness and cortical bone material stiffness, cortical bone thickness and rib cross-sectional width, and rib cross-sectional width and cortical bone material stiffness. For all remaining parameters, the top three interaction effects were with rib cortical bone thickness, rib cortical bone material stiffness, and rib cross-sectional width.

## 4 Discussion

This study used a variance-based parametric sensitivity analysis to identify the most influential property variations in the human thorax for SHBM NFR2+ rib fracture prediction in frontal and near-side impacts.

### 4.1 Findings

In both impacts, parameters representing rib properties (rib cortical bone thickness, rib cortical bone material stiffness, and rib cross-sectional width) had the greatest influence on the NFR2+ predictions. (See Figures 6, 7; Supplementary Table SA1). Rib fracture risk increased with reduced cortical bone thickness, reduced cortical bone material stiffness, and reduced cross-sectional width. The first two results are in line with findings from previous HBM studies, which reduced these properties in HBMs to create average representations of older individuals (Kent et al., 2005; Schoell et al., 2015; von Kleeck et al., 2022). For reduced cross-sectional width, increased fracture risk is in line with findings from single rib fracture modelling, where decreased cross-sectional width in a region of the rib increased element damage (element fracture criteria) in that region (Rampersadh et al., 2022). Further, rib material properties and rib cross-sectional dimensions have previously been identified as highly influential parameters for the overall structural responses and strain in parameter studies using HBM ribs under single rib-bending conditions (Fleischmann et al., 2020; Iraeus et al., 2020). In physical impact experiments with human ribs, the regression model with the highest explanatory power for peak force measured before rib fracture was based on a combination of age and the Whole Bone Strength Index (Agnew et al., 2018). The Whole Bone Strength Index is section modulus divided by the rib length. Since rib cortical bone material properties generally degrade with increasing age, the age factor in that regression model may serve as a proxy for degrading material properties. Furthermore, among single predictors for peak force, rib cross-sectional measures such as maximum and minimum area moment of inertia, and total- and cortical bone area have the greatest explanatory power (Agnew et al., 2018). These cross-sectional measurements are directly related to rib width and the cortical bone thickness around the rib cross-sections. These observations indicate that the results from the present study correlate with physical human rib findings.

Compared to the top three parameters, the other parameters had a much smaller influence on the results. The ribcage shape parameters (Ribcage shape PCs 1–6) determined the overall shape of the ribs and ribcage, which influenced how the external loading (from, e.g., the seatbelt and airbags) was distributed among individual ribs (Larsson et al., 2022a). Of these parameters, PCs 1 and 2 (Supplementary Table SA1) were the most influential. However, the greatest magnitude scaling coordinate values for these parameters had the greatest effects on the risk prediction. These values correspond to individuals which have ribcage shape variations (PC scores) above 1.8 SDs away from the average, and thus have less weight in the sensitivity analysis. In other words, in comparison to the top three parameters, the global ribcage shape variability is not highly influential for the rib fracture outcome for most individuals.

Moreover, the soft tissue material parameters were the least influential parameters in both impact configurations. Parameter ranges were set to correspond to ranges of test results from different tests. Therefore, these three parameters were assigned uniform distributions as there is insufficient data to determine distributions, and to determine if any one of the test results are more common. As a result, the effect of these parameters was effectively increased, since, in the sensitivity analysis, the same weighting was applied for results obtained for high-magnitude scaling coordinate values as for the low values. Still, their influence was comparatively low. The parametric adipose and muscle tissue material models were compared to compressive test results of adipose tissue (Comley and Fleck, 2012), and cross-fiber muscle tissue (Böl et al., 2012; Zhai and Chen, 2019) in Supplementary Figure SA2. While the adipose tissue model shows a substantial sensitivity to loading rate, the parametrized Lanzl et al. (2021) muscle model does not. The soft tissues in the HBM torso act as layers that transmit external loading to (or within, in the case of the intercostal muscle) the ribcage, but the rib fracture risk was only marginally affected by variability in their material properties within the current ranges. It should be noted that due to the rate dependency, the soft tissue material models have the potential to be more influential for other loading rates. Additional test data characterizing human soft tissue behavior in vehicle crash loading is needed.

As noted, the three most influential parameters were found to be those affecting the material properties and cross-sectional measures of the ribs themselves. Their predominant influence can be explained by linear elastic beam theory. In both impacts, the ribs were generally deformed through bending. The flexural rigidity, or bending resistance, of a beam structure is the product of Young’s modulus and the second area moment of inertia for the axis of bending. Here, Young’s modulus was directly influenced by rib cortical bone material, and the second area moment of inertia was influenced by the ribs’ cortical bone thickness and cross-sectional width. (Rib cross-sectional height also influences the area moment of inertia, but for the current axis of bending, the width of the rib is the dominating dimension.) The rib trabecular bone material is enclosed within the cortical shell and is approximately two to three orders of magnitude softer than the cortical bone, and thus had a smaller influence on the flexural rigidity of the ribs. Of the remaining parameters—for features external to the ribs—those for adipose tissue and skeletal muscle tissue contributed to the way the external loading was distributed, and those for intercostal muscle, costal cartilage, and overall ribcage shape contributed to the loading among the individual ribs. However, the most influential parameters were those most closely linked to controlling the resistance to deformation of the ribs themselves. That is, among the studied factors, those affecting the flexural rigidity of the ribs are most relevant for the occupant rib fracture risk predictions.

### 4.2 Ranges for the most influential parameters

For realistic sensitivity analysis results, it is important that the parameters and their ranges correspond to the extent of variability existing in the population. For the three most influential parameters, some assumptions about their variability were made.

**FIGURE 8:**
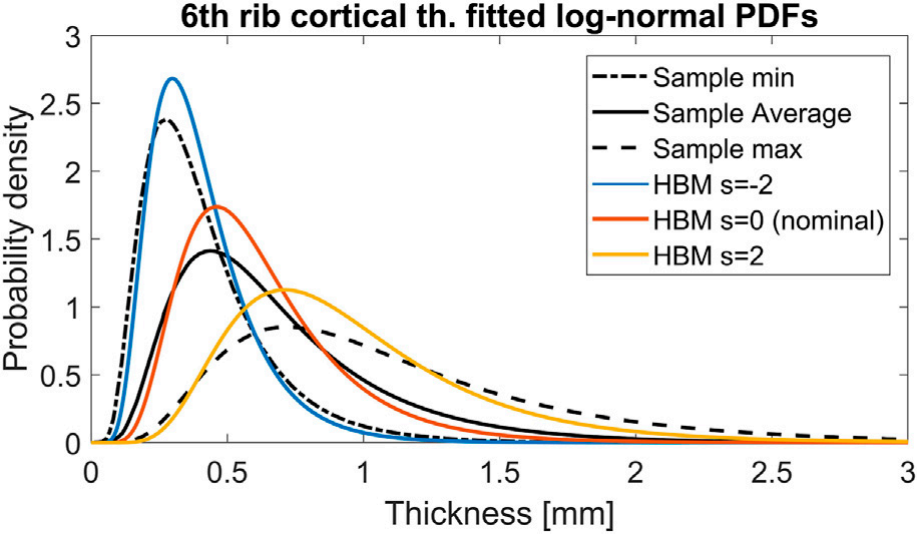
Probability density functions of sixth rib cortical bone thickness. Black curves correspond to the overall thinnest, average, and thickest individual bones in the Holcombe et al. (2019) n = 33 sample. Blue, orange, and yellow curves demonstrate the distribution of cortical bone thickness in the SHBM for s = −2, s = 0 and s = 2, respectively.

For rib cortical bone thickness variability, the scaling expression used (Eq. 2; Table 1) varied the thickness around the nominal thickness value at all nodes in all 24 ribs, assuming that the cortical bone thickness variability identified in sixth-level ribs is representative for all rib levels. For the SHBM sixth rib, distributions fitted to the cortical bone thickness for different levels of the scaling coordinate are shown in Figure 8, together with the overall thinnest, thickest, and average thickness distributions from the Holcombe et al. (2019) sample used to define the scaling ranges. As shown in the figure, the used scaling range resulted in a range of SHBM sixth level rib bone thickness variability that was similar to the extent of variability in the sample. Holcombe and Derstine (2022) recently presented SDs of rib cortical bone thickness at different measurement sites in rib levels 2 to 11, obtained from a sample comprising 240 males and females aged 20–90 years. The Holcombe and Derstine (2022) SD of rib cortical bone thickness, averaged for each rib level, was 0.24 mm for sixth level ribs and was similar in other rib levels (0.21–0.24 mm), indicating that the influence of cortical bone thickness variability on NFR2+ is not inflated by applying too-large thickness variations in the SHBM ribs.

The rib cross section width scaling of the SHBM ribs used a range based on sixth-level rib measurements, corresponding to a rib width SD of 1.35 mm (Eq. 3; Table 1). Kindig (2009) reported rib width SDs from rib levels 2 through 10 from nine PMHS’ (two female). The largest width SD of 2.3 mm was reported from the vertebral end of rib level 2, and the smallest SD of 0.5 mm at the posterolateral region of rib level 7. Averaged along the rib length, the Kindig rib width SDs for the different rib levels was 0.9–1.2 mm. Mohr et al. (2007) reported rib width SDs, averaged over the rib length, ranging from 2.1 mm (rib level 8) to 1.7 mm (rib level 7) from measurements of ribs at levels 3 through 9 in eight PMHS’ (five males and four females). Compared to Kindig and Mohr et al.‘s rib width SDs of 0.9–1.2 mm and 1.7–2.1 mm, respectively, the SD of 1.35 mm used for the SHBM rib widths appear reasonable. The influence of rib cross-sectional dimensions on HBM rib fracture risk predictions highlight the importance of detailed human reference data along the length of the ribs, including inter-individual variability.

**FIGURE 9:**
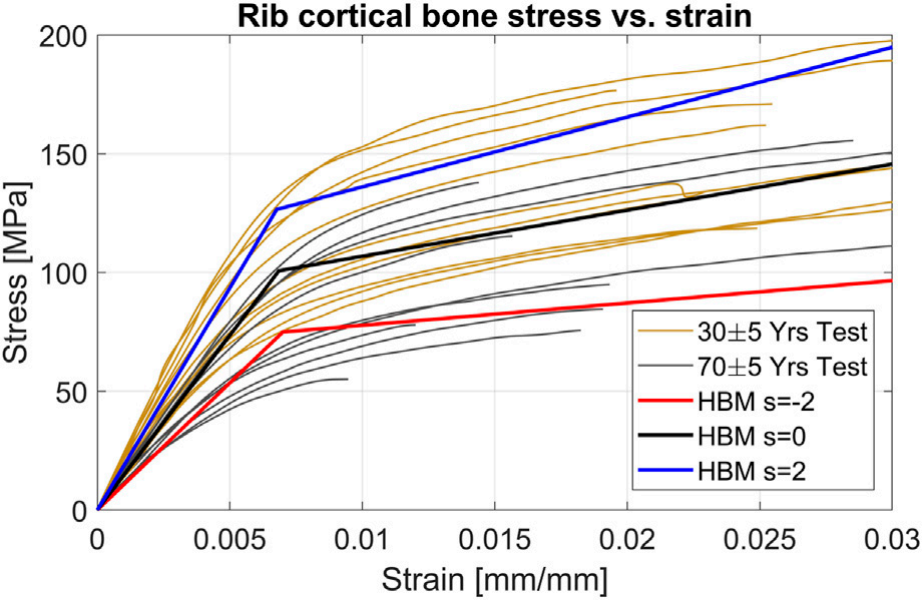
Individual test results from rib cortical bone tensile testing for younger (30 ± 5 years) and older (70 ± 5 years) subjects, together with scaled HBM cortical bone material curves.

The parametrization of the rib cortical bone material scaled several parameters together to achieve a bi-linear material response representing overall stiffer and softer material characteristics. The scaling ranges were based on published standard deviations of material parameters for the material model used in the SHBM ribs (Iraeus et al., 2020), based on test results from 12 individuals of various ages (Kemper et al., 2005; Kemper et al., 2007). Therefore, the SD used in this study was influenced by the effect of aging. We can visually compare the scaling range used to more recent test results from different individuals (Katzenberger et al., 2020). Figure 9 shows the average (s = 0) and ±2*SDs (s = ±2) for the HBM rib material, together with individual test results from younger (30 ± 5 years old) and older (70 ± 5 years old) subjects. While material from the younger subjects tends to have higher stresses (for a given level of strain) than that from the older subjects, the range of variability within each age group is comparable to the variability ranges used for the SHBM in the current study. As the SHBM range of rib material properties appear centered between the younger and older subject results, the range of variability used here roughly corresponds to the range of variability in material properties that can be expected for subjects around 50 years of age. As the rib fracture risk was lower for stiffer material and higher for softer materials (Supplementary Table SA1), it is likely that the sensitivity to variability in cortical bone material is age-dependent. If variability were modeled around the younger subjects’ average, there would be an overall decrease in predicted NFR2+ risk. As the rib fracture risk cannot go below 0%, the range of resulting risks can potentially be compressed by this lower bound. Similarly, modeling variability around the older subjects’ average might produce several predictions at the 100% risk level. Hence, the age trends in rib cortical bone material properties should be considered when variability is considered within a certain subpopulation, such as elderly occupants. Further, as judged by visual comparison in Figure 9, the method of scaling rib cortical bone material parameters together corresponds well to how stress-strain responses tend to differ between individual samples. That is, an increased Young’s modulus (initial slope of curves), is related to an increased yield stress (stress level where the curve bends away from the initial linear trend in the test results, kink in material model) and increased plastic modulus (the later slope of the curve). A strong correlation between Young’s modulus and yield stress, but not for plastic modulus has been reported from analysis of individual results from 12 subjects (Iraeus et al., 2020). More detailed parameter sensitivity analysis can determine if plastic modulus variability needs to be considered separately in future work.

### 4.3 Parametric sensitivity analysis

A variance-based parametric sensitivity analysis method was used for the current study. The method considered the distribution of each parameter within the population. Considering the distributions is important, since small effects from common parameter values might have an overall greater contribution to rib fracture outcomes than large effects from extreme, but rare, parameter values when outcomes are aggregated across a population. Further, this method was able to demonstrate the existence of interaction effects between different parameters. However, the sensitivity indices were calculated using an approximative method based on the assumption that the function being analyzed can be decomposed according to the M-DRM (Eq. 9). It has been shown that this method can compute the sensitivity indices with accuracy similar to state-of-the art Monte Carlo methods for several different functions, provided enough integration points are used (Zhang and Pandey, 2014). Here, we used a five-point Gauss-Legendre quadrature, which integrates ninth-degree polynomials exactly. Analysis with three-point integration resulted in similar sensitivity indices in both impacts and also identified as most influential the same three parameters. Still, how well the M-DRM assumption holds for HBM occupant rib fracture risk is not known, and thus the sensitivity analysis results should be interpreted as indicative. Evaluating the sensitivity indices for the rib fracture risk (approximately 6 h per function evaluation) with Monte Carlo-based computations was not feasible, due to the time cost that would have been required. Previous studies investigating HBM rib fracture sensitivity to parameters have used factorial analysis, where only a few parameters have been changed in a few steps (Kent et al., 2005; Schoell et al., 2015; von Kleeck et al., 2022). A full factorial analysis with three parameter levels (high, mid, and low) and the 15 parameters included in the current study would require over 14.3 million (3^15^) function evaluations. Including only two levels for the parameters will still require about 32.8 thousand (2^15^) function evaluations. Thus, the currently used method was a practical choice, but its accuracy for the current use case should be evaluated in future work.

### 4.4 Limitations and future work

There are several limitations with this study. First, only an average male HBM, in terms of height and weight, was used. The choice of HBM was based on previous validation of rib strain and rib strain-based rib fracture risk predictions (Iraeus and Pipkorn, 2019; Pipkorn et al., 2019). In these validations v.9 of SHBM was used. Updates for v.10 included new thoracic soft tissue meshes, including separate skeletal muscle and adipose tissue layers and a new pelvis model. The ribcage model, including intercostal muscles was kept from v.9 (Pipkorn et al., 2021). Parameter variability also exists among individuals in other subpopulations (such as small females or large males). For a given impact scenario, the amount and location of loading to the torso can change due to changes in body mass and height. However, the parameters identified as influential here, related to how the ribs resist deformation due to external loading, will likely remain influential even for other subpopulations. There is a possibility that the influence of soft tissue parameters increases if the relative volume of soft tissues increases (such as for obese occupants), which should be investigated in future work.

Secondly, only two impact conditions were considered in the current study; both cases were set up so that the average HBM obtained close to 50% risk of NFR2+. In real life, crashes occur in a wide range of angles and velocities. For both lower and higher impact velocities, the relative influence of the parameters would decrease. For very low impact speeds, the rib fracture risk could be 0% regardless of parameter settings, while it could become saturated at 100% for some or all parameter settings at higher impact speeds. The two impact scenarios used represent impact conditions with a high risk of occupant rib fractures in real-world crashes (Pipkorn et al., 2020), and represent two different modes of loading to the ribs. In a frontal impact, the loading from the seatbelt to the chest tends to bend the anterior ends of the ribs towards the spine, thus resulting in tensile strains on the cutaneous side of the ribs. In a side impact, the intruding side structure and airbag instead apply loading to the lateral region of the ribs, causing that region to bend inwards, resulting in tensile strains at the pleural side of the loaded ribs. Despite the different rib loading modes, the same parameters were found to be the most influential, which indicates that the results found here should be robust over a range of impact directions. It is possible that ribcage shape variability can have a larger influence under other external conditions, such as scenarios in which the ribcage either comes into contact, or not, with a vehicle interior component depending on ribcage dimensions. Further evaluations using different boundary conditions for thoracic loading are needed to confirm the general validity of the results.

Third, while the parameter study performed here is extensive, it is not exhaustive. The parameters included were limited to entities already represented in the HBM. For example, the rib cortical bone is modeled by shell elements with varying thickness. The elements represent homogeneous material throughout this thickness, while rib cortical bone has an inhomogeneous structure: individuals can, for example, have varying degrees of intra-cortical porosity (Agnew and Stout, 2012). These pores and other local microstructure properties can potentially serve as sources of stress concentration during loading and can thus be highly relevant for the individual rib fracture risk. Such details are, however, beyond the modeling capacity of current full-body HBMs for occupant impact simulations. Further, some entities present in the HBM thorax were not parametrized, such as the thoracic spine or the soft internal thoracic organs. From PMHS chest impact experiments with and without internal thoracic organs, it is known that 30%–40% of the thorax stiffness can be attributed to the internal organs (Kent, 2008; Murach et al., 2018). The thoracic internal organs are modeled by a lumped representation in the SHBM, for which no variability ranges could be identified. Further evaluations with more detailed rib or thoracic models may lead to a better understanding of how variability in bone structure and soft thoracic organs contributes to population rib fracture outcomes.

Fourth, the morphing procedure applied for some parameters altered the mesh and thereby the mesh quality. A reduced mesh quality increases the risk of numerical artifacts that can influence the results. However, in the current work, morphed elements passed in-house mesh quality criteria for the HBM, indicating a low risk for numerical artifacts. Further investigating the effect of improved element quality requires re-meshing. In that case, it is unknown if any changed results are due to the new mesh or due to parameter changes.

### 4.5 Outlook

The results from this study showed that rib cortical bone thickness, rib cross-sectional width, and rib material properties were the most influential parameters for HBM rib fracture risk predictions. These findings can aid the selection of model parameters in future HBM studies. For studies aiming to model the distribution of occupant rib fracture outcomes, including the population variability of the three most influential factors will likely result in a more realistic estimation of the injury distribution, while keeping the number of model parameters low. As individual variability exists for humans of different sex, age, height, and weight, the validity of rib fracture distributions computed in studies with morphed HBMs, representing occupants of different subpopulations, can be improved by including the important parameters identified here. HBMs that reflect both global and local variability among different occupants can be used to develop vehicles and safety systems with reduced rib fracture risks for all occupants.

## 5 Conclusion

Out of 15 evaluated structural and material factors, the greatest influence on predicted rib fracture risk were found for rib cortical bone thickness, rib cortical bone material properties, and rib cross-sectional width.

For rib fracture risk analysis with HBMs for the population of vehicle occupants, it is recommended that the variability in rib cortical bone thickness, rib cortical bone material properties, and rib cross sectional width be considered.

## Data Availability

The original contributions presented in the study are included in the article/Supplementary Material, further inquiries can be directed to the corresponding author.
