# Supplementary material for: Influences of human thorax variability on population rib fracture risk prediction using human body models
Source: Front Bioeng Biotechnol. 2023 Mar 23;11:1154272. doi: 10.3389/fbioe.2023.1154272 (PMC10078960; doi:10.3389/fbioe.2023.1154272)
Supplement: Supplementary file 1 [file DataSheet1.docx]

Table SA1. For each included parameter: ID (PID), name, assumed distribution, NFR2+ risk [%] for each simulation at each evaluated scaling coordinate (scaled Gauss point), and the range of risks obtained.

| PID | Parameter Name | Assumed distribution | $s=-1.81$ | $s=-1.08$ | $s=0$ | $s=1.08$ | $s=1.81$ | Range |
| --- | --- | --- | --- | --- | --- | --- | --- | --- |
| Frontal impact | | | | | | | | |
| 1 | Ribcage shape PC 1 | Normal | 64.0 | 61.0 | 50.8 | 76.4 | 77.0 | 26.2 |
| 2 | Ribcage shape PC 2 | Normal | 63.5 | 59.3 | 50.8 | 62.9 | 91.6 | 40.8 |
| 3 | Ribcage shape PC 3 | Normal | 54.4 | 52.1 | 50.8 | 49.4 | 51.5 | 5.0 |
| 4 | Ribcage shape PC 4 | Normal | 56.6 | 54.4 | 50.8 | 59.5 | 53.8 | 8.7 |
| 5 | Ribcage shape PC 5 | Normal | 49.5 | 47.0 | 50.8 | 56.4 | 59.2 | 12.3 |
| 6 | Ribcage shape PC 6 | Normal | 58.2 | 55.7 | 50.8 | 56.4 | 54.9 | 7.5 |
| 7 | Rib cortical bone thickness | Normal | 99.8 | 91.8 | 50.8 | 12.5 | 5.2 | 94.6 |
| 8 | Rib cross-sectional width | Normal | 93.9 | 84.0 | 50.8 | 35.8 | 31.9 | 62.0 |
| 9 | Rib cross-sectional height | Normal | 78.2 | 64.0 | 50.8 | 51.5 | 55.1 | 27.4 |
| 10 | Rib cortical bone material | Normal | 98.5 | 84.6 | 50.8 | 22.8 | 12.3 | 86.3 |
| 11 | Rib trabecular bone material | Normal | 72.7 | 54.6 | 50.8 | 53.0 | 49.4 | 23.3 |
| 12 | Costal cartilage modulus | Log-normal | 61.9 | 60.7 | 50.8 | 46.4 | 50.5 | 15.5 |
| 13 | Torso adipose tissue material | Uniform | 62.1 | 58.1 | 50.8 | 51.5 | 49.9 | 12.2 |
| 14 | Skeletal muscle tissue material | Uniform | 54.3 | 56.1 | 50.8 | 53.1 | 50.7 | 5.4 |
| 15 | Intercostal muscle material | Uniform | 50.5 | 54.0 | 50.8 | 52.3 | 56.1 | 5.6 |
| Near-side impact | | | | | | | | |
| 1 | Ribcage shape PC 1 | Normal | 26.0 | 45.3 | 51.0 | 72.8 | 76.8 | 50.9 |
| 2 | Ribcage shape PC 2 | Normal | 89.9 | 78.3 | 51.0 | 39.2 | 42.2 | 50.6 |
| 3 | Ribcage shape PC 3 | Normal | 39.1 | 45.3 | 51.0 | 66.0 | 80.9 | 41.8 |
| 4 | Ribcage shape PC 4 | Normal | 52.0 | 47.9 | 51.0 | 51.6 | 47.9 | 4.2 |
| 5 | Ribcage shape PC 5 | Normal | 37.9 | 42.7 | 51.0 | 61.9 | 77.6 | 39.8 |
| 6 | Ribcage shape PC 6 | Normal | 54.6 | 53.3 | 51.0 | 76.3 | 72.2 | 25.3 |
| 7 | Rib cortical bone thickness | Normal | 99.9 | 95.9 | 51.0 | 8.0 | 0.8 | 99.1 |
| 8 | Rib cross-sectional width | Normal | 99.7 | 93.5 | 51.0 | 29.9 | 14.9 | 84.9 |
| 9 | Rib cross-sectional height | Normal | 36.8 | 50.9 | 51.0 | 75.3 | 90.6 | 53.8 |
| 10 | Rib cortical bone material | Normal | 99.6 | 92.6 | 51.0 | 22.4 | 10.5 | 89.1 |
| 11 | Rib trabecular bone material | Normal | 79.7 | 64.9 | 51.0 | 43.9 | 40.6 | 39.1 |
| 12 | Costal cartilage modulus | Log-normal | 48.4 | 57.6 | 51.0 | 53.8 | 59.1 | 10.6 |
| 13 | Torso adipose tissue material | Uniform | 31.9 | 42.4 | 51.0 | 60.6 | 54.2 | 28.7 |
| 14 | Skeletal muscle tissue material | Uniform | 60.7 | 59.6 | 51.0 | 54.0 | 55.2 | 9.7 |
| 15 | Intercostal muscle material | Uniform | 59.3 | 57.0 | 51.0 | 62.5 | 56.8 | 11.5 |


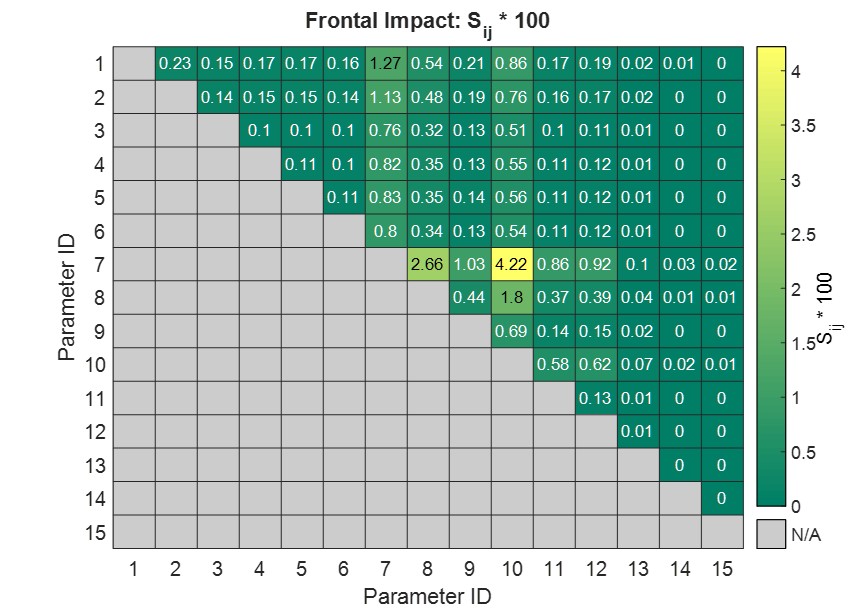


Figure SA1. Frontal impact, second-order sensitivity indices, multiplied by a factor of 100. Parameter IDs are defined in Table A1.


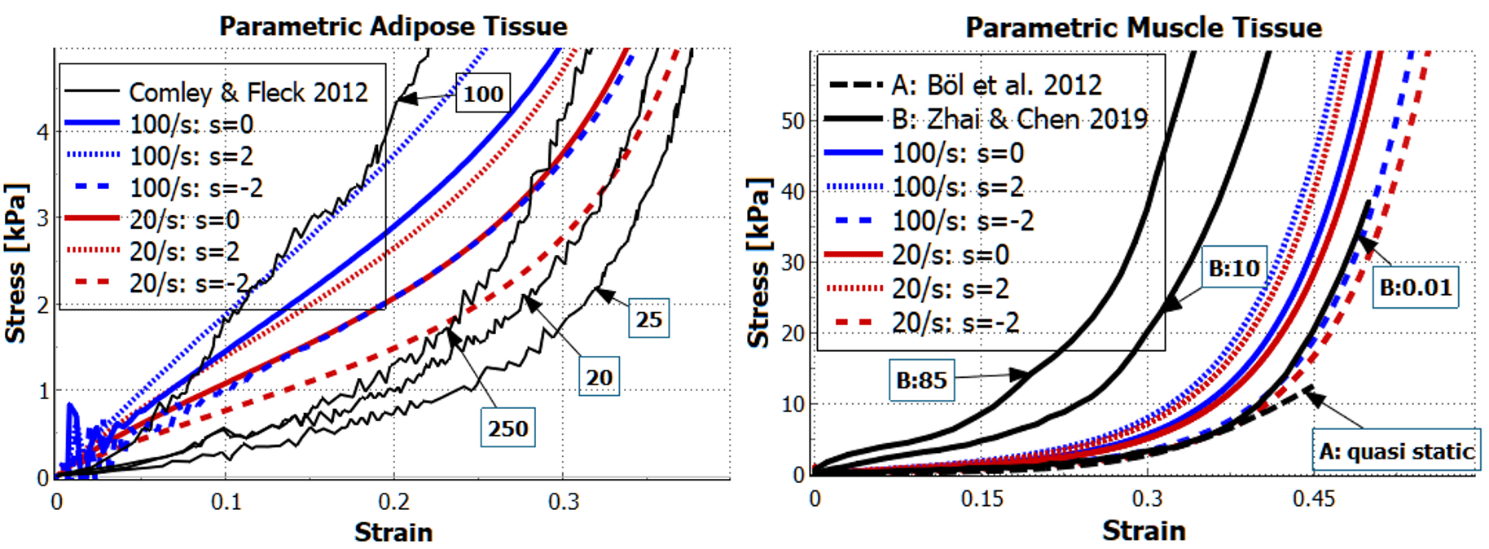


Figure SA2. Left: parametric adipose tissue compressive stress vs. strain with strain rates 100/s (blue) and 20/s (red) for different values of scaling coordinate s. Compressive adipose tissue test results (black) for different strain rates (indicated by arrows and small text boxes). Right: parametric muscle tissue compressive stress vs. strain with strain rates 100/s (blue) and 20/s (red) for different values of scaling coordinate s. Compressive test results for rabbit (A, dashed black) and porcine (B, solid) for different strain rates (indicated by arrows and small text boxes).
